# Complete Lifestyle Medicine Intervention Program–Ontario: Implementation Protocol for a Rural Study

**DOI:** 10.2196/59179

**Published:** 2024-12-31

**Authors:** Kush Patel, Lisa Allen, Karine Boucher, Michelle Fedele, Debbie Fong, Sangeeta Kumar, Deanna Lavigne, Elisa Marin-Couture, Magdalena Partyka-Sitnik, Nicole Rietze, Jenna Smith-Turchyn, Mylene Juneau, Caroline Rhéaume

**Affiliations:** 1 Northern Ontario School of Medicine University Sudbury, ON Canada; 2 Parry Sound Local Education Group Parry Sound, ON Canada; 3 College of Dietitians of Ontario Toronto, ON Canada; 4 College of Traditional Chinese Medicine Practitioners and Acupuncturists of Ontario Thornhill, ON Canada; 5 Deanna Lavigne Kinesiology Huntsville, ON Canada; 6 Department of Kinesiology Faculty of Medicine Université Laval Québec, QC Canada; 7 West Parry Sound Health Center Parry Sound, ON Canada; 8 School of Rehabilitation McMaster University Hamilton, ON Canada; 9 Department of Family Medicine and Emergency Medicine Faculty of Medicine Université Laval Québec, QC Canada

**Keywords:** chronic disease, nutrition, exercise, sleep, relationships, stress reduction, self-compassion, risky substance use, holistic medicine, whole health, implementation, lifestyle medicine, rural medicine, web-based platform, substance use, feasibility, wellness, barriers, opportunities

## Abstract

**Background:**

Sedentary lifestyles, poor nutritional choices, inadequate sleep, risky substance use, limited social connections, and high stress contribute to the growing prevalence of chronic diseases. Lifestyle medicine, emphasizing therapeutic lifestyle changes for prevention and treatment, has demonstrated effectiveness but remains underutilized in clinical settings. The Complete Lifestyle Medicine Intervention Program–Ontario (CLIP-ON) was developed to educate the rural population of Northern Ontario in lifestyle medicine to improve health outcomes and engagement.

**Objective:**

This study evaluates the implementation and effectiveness of the CLIP-ON program for patients with chronic diseases in the Parry Sound area, focusing on lifestyle behaviors, health outcomes, enrollment, retention rates, and interdisciplinary team engagement.

**Methods:**

This observational cohort study guided by the RE-AIM framework (Reach, Effectiveness, Adoption, Implementation, and Maintenance) includes pre- and postintervention assessments from participants and health care providers. A hybrid type II mixed methods design evaluates the intervention’s effectiveness and implementation process in real-world settings through quantitative and qualitative data collection. CLIP-ON is tailored to the residents of the Parry Sound catchment area in Northern Ontario. Participants (≥18 years old) with chronic conditions such as prediabetes, type II diabetes, systemic hypertension, cardiovascular vascular disease, dyslipidemia, or high BMI (≥25) will be recruited through self-referral or provider referral. Approximately 10 participants per cohort will be enrolled in the CLIP-ON program, consisting of 22 weeks of weekly group sessions and monthly individual consultations with physicians, health coaches, kinesiologists, and registered dieticians either in person or through a web-based platform. CLIP-ON will cover the 6 pillars of lifestyle medicine through 14 group sessions followed by an 8-week supervised exercise program. Anthropometric and cardiometabolic variables will be measured before and after the program. Participants will be surveyed on lifestyle habits, wellness, perceived barriers, and program satisfaction at 3 and 6 months. Focus groups and dropout interviews with participants (n=10 per cohort) and providers (n=6 per cohort) will guide program adaptations. Quantitative and qualitative data collected at baseline and follow-up will assess the program’s implementation and identify barriers and opportunities for improvement.

**Results:**

This study was approved by the Laurentian University Research Ethics Board (6021397) on July 6, 2023. The first cohort was enrolled in late 2023 and is still under evaluation. The second cohort began in mid-2024, and data collection is currently underway. A mixed methods analysis will be used at enrollment, program completion (22 weeks), and follow-up (6 months after program completion). Focus groups assessing the program’s effectiveness and implementation will take place after the 22-week intervention. Data will be analyzed in early 2025.

**Conclusions:**

This protocol provides insights into the implementation of this lifestyle medicine program and its impact on participants’ health. The findings will guide future advancements and establish a scalable model for other communities.

**Trial Registration:**

ClinicalTrials.gov NCT06192251; https://clinicaltrials.gov/study/NCT06192251

**International Registered Report Identifier (IRRID):**

DERR1-10.2196/59179

## Introduction

### Background

Chronic diseases, also known as noncommunicable diseases (NCDs) such as cancer, cardiovascular disease, cerebrovascular disease, and diabetes, are among the leading causes of death in Canada, with their prevalence steadily rising [1]. Hypertension, the leading global risk factor for death and disability, remains uncontrolled in more than 50% of patients [2]. NCDs are responsible for over 74% of global deaths annually [2-4], significantly affecting vulnerable and low-income populations [5-7]. Despite clear benefits from improved lifestyle choices, including better nutrition, regular physical activity, and stress reduction, there remains limited research on the long-term sustainability of these changes and the role of physician intervention [5,7-9].

Sedentary lifestyles, poor nutritional choices, and increased stress play significant roles in chronic disease development [7,8,10], with type II diabetes affecting over 10% of the population worldwide [11]. Type II diabetes, primarily linked to cardiovascular disease, contributes to over 1 million deaths annually [7,12-14]. Chronic disease–related health care costs in Canada account for more than US $136 billion annually [15], emphasizing the need for lifestyle interventions to reduce this burden [1,14].

Lifestyle medicine is an interdisciplinary medical specialty that focuses on 6 key pillars such as dietary changes, regular physical activity, stress management, restorative sleep, positive social connection, and avoidance of risky substances such as alcohol and tobacco has been shown to improve outcomes in chronic conditions [16-18]. Moreover, with its patient-centered focus [6,19-21], lifestyle medicine addresses the root causes of disease, aligning with the P4 (Preventive, Predictive, Personalized, and Participatory) medicine approach [6,22,23]. Achieving optimal health through a maintained commitment to lifestyle medicine has been shown to reverse many stages of chronic disease, reduce hospitalization and hospital costs, improve chronic disease management, and promote better health outcomes [4,5,14,24-28].

Studies indicate that the adoption of evidence-based lifestyle medicine practices has gained traction, with numerous programs demonstrating effectiveness in improving health outcomes and promoting sustainable behavior change [9,29-31]. Other studies support the adoption of evidence-based lifestyle medicine practices across North America [9,20,32,33]. Such evidence highlights the potential for lifestyle medicine to be integrated into routine health care, paving the way for broader acceptance and implementation in various clinical environments.

However, despite these promising outcomes, effective implementation of lifestyle medicine in clinical settings remains challenging. A strong patient-provider collaborative relationship is essential for achieving adherence to treatment plans and informed health care decision-making [34]. The physician’s role as a health coach is critical in this process, underscoring the importance of training clinicians in lifestyle medicine practices [5,12,16,35]. Yet, many physicians report a lack of confidence and skills in delivering lifestyle medicine effectively [20], especially in rural areas. During the COVID-19 pandemic, virtual and digital platforms, such as lifestyle management tools, were developed to support patients [10,36], which has proven to be crucial for equitable health care access [16,35]. These tools hold the potential to address barriers in underserved areas where health care resources are limited, particularly in rural settings.

In this context, it becomes essential to evaluate the real-world implementation of lifestyle medicine to identify both successes and challenges, ensuring that lifestyle medicine can be effectively integrated into routine health care. This is especially relevant in rural areas, where health care access is limited, and the burden of chronic disease is high. Parry Sound, a rural community in Northern Ontario with a population of 6879 and a catchment area of 42,824 (including 10% Indigenous residents) [26,29,36,37], experiences disproportionately high rates of diabetes (8%) and hypertension (20%) compared with the provincial average [27]. These factors make it an ideal location for integrating lifestyle medicine into care. Incorporating traditional Indigenous teachings, which emphasize the interconnectedness of mind, body, spirit, and emotions, can bridge gaps between traditional Western medicine and Indigenous Healing Practices and improve population health outcomes [28,36,38].

Inspired by the New York Lifestyle Medicine program [36,38], in 2023 our medical and research team, in collaboration with the health care community in Parry Sound, developed an innovative and whole health program named the Complete Lifestyle medicine Intervention Program, Ontario (CLIP-ON). These comprehensive interdisciplinary aims are to educate participants on the 6 pillars of lifestyle medicine and inform them about their integration into daily life to mitigate chronic disease and enhance overall health. To facilitate the implementation of lifestyle medicine practices, various models and methodologies have been proposed [39]. Among these, the RE-AIM framework (Reach, Effectiveness, Adoption, Implementation, and Maintenance) serves as a valuable tool for evaluating the impact of lifestyle medicine interventions in diverse settings [40,41]. To the best of our knowledge, this is the first program in lifestyle medicine in a rural area in Ontario. Our overarching hypothesis is that the implementation of CLIP-ON will significantly improve lifestyle behavior, health outcomes, and participant engagement, with feedback from participants and health care providers informing real-time program improvements.

### Primary Objective

To evaluate the implementation and effectiveness of the comprehensive web-based platform and in-person CLIP-ON program for patients with chronic disease in the Parry Sound area, focusing on its impact on lifestyle behaviors, health outcomes including cardiometabolic parameters, and participants’ engagement.

### Secondary Objectives

The secondary objectives of this study are (1) to assess the reach and adoption of the CLIP-ON program by evaluating participant enrollment, and retention rates and (2) to gather and analyze direct feedback from participants and health care providers to inform real-time program improvements and enhance the program’s overall effectiveness.

## Methods

### Design

This protocol outlines an observational cohort study guided by the RE-AIM framework (Reach, Effectiveness, Adoption, Implementation, and Maintenance) and includes pre- and postintervention assessments from participants and health care providers (RE-AIM milestones Multimedia Appendix 1). A hybrid design type II mixed methods approach [42,43] will be used to simultaneously evaluate both the effectiveness of the CLIP-ON and its implementation process in a real-world setting. This will involve the collection and analysis of both quantitative and qualitative data.

**Figure 1:**
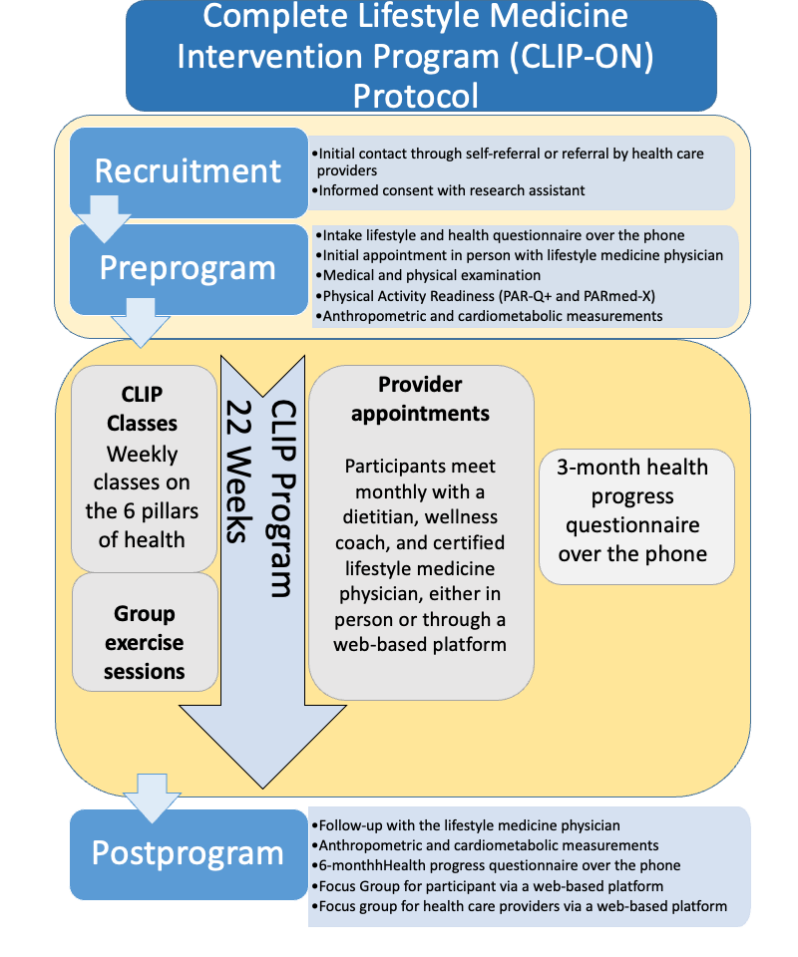
CLIP-ON protocol. The intake lifestyle and health progress questionnaire was inspired by the Lifestyle Assessment Short Form [46], the 36-Item Short Form Health Survey, and the Patient Health Questionnaire-9 [47,48]. The Physical Activity Readiness Questionnaire (PAR-Q+) [44] and PARmed-X [45] were used to measure physical activity readiness. The 6 pillars of health inspired by the American College of Lifestyle Medicine are nutrition, sleep, relationships, physical activity, risky substance use, and stress management [18]. Three- and 6-month health progress questionnaires were inspired by the questions used in the New York City Health [38,49,50], the Hospital lifestyle medicine program, and the Complete Health Improvement Program lifestyle medicine program at Vanderbilt University [51,52].

**Figure 2:**
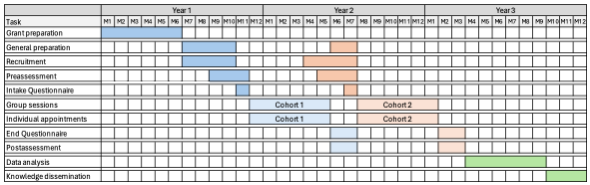
Timeline. It illustrates the key tasks throughout the study timeline. The first year focused on grant preparation, recruitment, preassessment, and intake questionnaires to prepare the first cohort. Year 2 emphasizes group sessions, individual appointments, end-of-program questionnaires, and postassessment for Cohort 1, while also initiating the same process for Cohort 2. Year 3 will primarily focus on data analysis and knowledge dissemination.

The CLIP-ON program is an interdisciplinary lifestyle medicine intervention delivered both virtually and in person, specifically tailored for residents of the Parry Sound catchment area in Northern Ontario. Within this district are 3 First Nation Communities (Wasauksing, Moose Deer Point, and Shawanaga First Nations), each of which has requested access to CLIP-ON. The study design is summarized in Figure 1, and the participant’s and health care providers’ measurements are presented in Table 1. Following informed consent, each participant will have an initial appointment with a certified lifestyle medicine physician at West Parry Sound Health Centre before starting the CLIP-ON program. Participants will complete the Physical Activity Readiness Questionnaire (PAR-Q+) by the Canadian Society of Exercise Physiology [44] to identify risk factors during moderate physical activity. A Physical Activity Readiness Medical Examination (PARmed-X) [45] will also be completed for participants who had potential medical complications from exercise according to their response to the PAR-Q+. During this visit, they will undergo a medical review and physical examination. They will also receive a requisition for blood work to establish their baseline cardiometabolic data. Each participant will complete a baseline (intake Lifestyle and health) questionnaire on Google Forms with the assistance of a trained research staff member over the phone. This preprogram questionnaire will collect demographic and baseline information about their lifestyle according to the 6 pillars of lifestyle medicine. Cardiometabolic variables will be measured before and after the CLIP-ON intervention. Participants will be surveyed by phone at 3- and 6-months regarding lifestyle habits, wellness, perceived barriers, and program satisfaction. At the end of the program, web-based platform (through Zoom) focus groups with participants and health care providers will be conducted to discuss their experiences and provide feedback for program development. Focus groups and dropout interviews with patients (approximately 10 per cohort, anticipating a drop-out rate of 10% based on similar studies [29], and providers (approximately 6 per cohort) will provide iterative feedback, enabling program refinement. The 2 cohorts are planned, with potential for expansion based on the available funding. The study timeline is illustrated in Figure 2.

**Table 1:** Participants and health care providers measurements.

Measurement	Time	Variables	Details
Intake Lifestyle and Health Participants Questionnaire	Baseline, 6 months, and 12 months	Food consumption, motivation and confidence, neighborhood food environment, physical activity, media use and screen time, substance use, sleep, health, behavior, and well-being, and socio-demographics	Administered through phone or online, inspired by inspired by the Lifestyle Assessment Short Form [46], the short form survey instrument SF-36^a^ and the PHQ-9^b^ [47,48].
Health progress questionnaire^c^	3 months and 6 months	Similar to the intake questionnaire but includes additional questions on social support, satisfaction with the program	Conducted by phone with a research assistant
Anthropometric measurements	Pre- and postprogram	Health, weight, BMI, and waist circumference	Measurements taken by health care providers
Hemodynamic measurements	Pre- and postprogram	Blood pressure and heart rate	Measurements taken by health care providers
Cardiometabolic measurements	Pre- and postprogram	Hemoglobin, ions (calcium, magnesium, phosphate, sodium, potassium), Fasting blood glucose, Glycated Hemoglobin, cholesterol Lipid panels (total cholesterol, high-density lipoprotein cholesterol, low-density lipoprotein cholesterol, plasma, triglycerides) creatine glomerular filtration rate, Urine test: microalbumin, and albumin-creatine ratio	Blood and urine tests collected for analysis
Health care provider questionnaire	Preprogram	Provider social demographics, credentials, practice location	Completed before program involvement
Participants focus group	End of program	Experiences, benefits, challenges, program feedback, their thoughts on lifestyle medicine pillars addressed, and program continuation	Virtual focus group using a semistructured script led by an independent researcher or assistant
Health care provider focus	End of program	Feedback on recruitment strategies, their experiences, their thoughts on the content and materials provided, challenges encountered, future sustainability, suggestions for future program implementation cohort	Virtual focus group using a semistructured script led by an independent researcher or assistant

^a^SF-36: 36-item Short Form Survey.

^b^PHQ-9: Patient Health Questionnaire-9.

^c^These questionnaires include questions used in the Complete Health Improvement Program (CHIP) lifestyle medicine program at Linda Loma University and the New York City Health and Hospital lifestyle medicine program [38,49]. In addition, the Warwick, -Edinburgh Mental Well-Being scale guided the inclusion of questions pertaining to mental health [51,52]. Together, these existing surveys allow both quantitative and qualitative elements to be included in this study.

Figure 1 demonstrates participant flow throughout the CLIP-ON study, a hybrid lifestyle medicine program designed for rural Northern Ontario. The program addresses 6 pillars of health: healthy nutrition, regular physical activity, restorative sleep, stress management, avoiding risky substance use, and fostering positive relationships. Patients could meet health care providers in person or through a web-based platform. Group sessions and exercise classes during the 22-week program were available in 3 formats: in person, through a web-based platform, or as recorded sessions for later access. Intake lifestyle and health progress questionnaire inspired by the Lifestyle Assessment Short Form [46], the short form survey instrument SF-36 and the PHQ-9 [47,48]. Physical Activity Readiness Questionnaire: PAR-Q+ [44] and PARmed-X [45]. Three- and 6-Month Health Progress Questionnaire inspired by the questions used in the New York City Health [38,49,50] and Hospital Lifestyle Medicine program and the CHIP lifestyle medicine program at Vanderbilt University [51,52].

### Participants

#### Inclusion Criteria

Adult participants (≥ 18 years) with chronic diseases such as prediabetes, type II diabetes mellitus, systemic hypertension, coronary heart disease, peripheral vascular disease, dyslipidemia, or health concerns related to excessive body weight (BMI≥25) will be recruited. Participants must reside in the Parry Sound, Ontario catchment area.

#### Exclusion Criteria

Participants with unstable medical conditions that prevent successful completion of program elements will be excluded. In addition, individuals who are unable to provide consent, do not meet the requirements of PARmed-X, and are unable to engage in a low-intensity, a professionally supervised exercise program will also be excluded.

### Recruitment

Participants will be recruited through self-referral or referral by health care providers. Multiple avenues will be pursued to enhance recruitment efforts and reach a diverse population. Posters will be distributed throughout the Parry Sound community in high-traffic public locations such as grocery stores, coffee shops, libraries, and various departments within the Parry Sound Community Hospital. Pamphlets will be made available at health care provider clinics both within and outside of the hospital, including the Family Health Team and nurse-practitioner-led clinics. In addition, both posters and pamphlets will be distributed to local health care providers, who will be encouraged to share on social media (Facebook and Instagram [Meta]) in accordance with the ethical agreement to maximize outreach to the target population. These comprehensive recruitment strategies aim to ensure saturation of the rural environment and ensure equal opportunities for eligible participants to join the program.

### Implementation Assessment

The CLIP-ON implementation assessment is guided by the RE-AIM framework which defines 5 dimensions: reach, effectiveness, adoption, implementation, and maintenance [40,41]. These dimensions will also help guide enhancements to the CLIP-ON program. Further details are provided in Multimedia Appendix 1.

### Description of the CLIP-ON Program

#### Overview

The CLIP-ON program will consist of 22 weeks of weekly group sessions held at the local Parry Sound Bobby Orr Community Centre and monthly individual consultations, either in person at West Parry Sound Health Center or virtually, with an interdisciplinary team including physicians, a health coach, and registered dieticians. Participants will be encouraged to attend all 22 weekly classes and exercise sessions in person. However, a web-based platform option will be available for instances when they are unable to attend. Both the group class sessions and exercise sessions will be recorded for participants to access later. Each participant will receive a cookbook, exercise bands appropriate for their fitness level, and US $14.22 gift cards for local grocery stores as compensation for their time in completing program surveys and participating in focus groups.

#### Fundamentals of Lifestyle Medicine Group Class

CLIP-ON will cover the 6 pillars of lifestyle medicine through 12-14 group sessions on the Fundamentals of Lifestyle Medicine followed by an 8-week supervised exercise program developed and supervised by a kinesiologist. Table 2 outlines the topics covered in these group classes with a brief description of each. These classes will address the importance of the human microbiome and nutrition, fitness, positive relationship integration, stress and sleep management, and navigation of substance use toxicity and addiction. Concurrently, participants will discuss goal setting and planning with their lifestyle medicine physician, health coach, and dietician to incorporate knowledge and skills developed through the classes into their lives while motivating their success in the program.

**Table 2:** Topics and descriptions of each Fundamentals of Lifestyle Medicine group class.

Class topic	Class description	Providers
Introduction	An overview of what lifestyle medicine is and small group discussions for participants to meet each other.	Lifestyle medicine physician
Microbiome	Mostly focused on the gut microbiome, what it does, how every pillar can help build and maintain it, and what happens if we do not.	Lifestyle medicine physician
Nutrition	Two classes, one focused on explaining the recommendations of Canada’s food guide, and another on discussing food preparation, practical cooking tips, and reading food labels.	Dietitian
Physical activity	Two Classes, one on the importance and benefits of physical activity and The Canadian Society of Exercise Physiologists’ recommendations, and another class on how to move safely.	Kinesiologist
Sleep	Two classes, one on the benefits of sleep and what happens if we do not get enough sleep, another class on how to build our sleep hygiene, and an overview of insomnia and Cognitive Behavioral Therapy for insomnia.	Lifestyle medicine physician
Stress	Two classes, one on the impact of stress and the different ways it can manifest itself in our lives, and another class on stress management techniques.	Health coach
Social connections and positive psychology	One class on the impact of social isolation and the Positive Emotion, Engagement, relationships, Meaning, and Accomplishment (PERMA) [53,54] model of positive psychology for happiness and fulfillment.	Lifestyle medicine physician
Relationships with ourselves and others	One class on the concepts of Mindful Self-Compassion was developed by Kristin Neff and Christopher Germer, and Nonviolent Communication was developed by Marshall B. Rosenberg.	Lifestyle medicine and health coach
Risky substances and addictions	One class on the impact of addictions, the risk factors that can lead to addictive behaviors, and how to build our own resilience.	Lifestyle medicine physician
Conclusion	Discussion of takeaways and habit-building tips.	Lifestyle medicine physician

#### Exercise Group Class

An 8-week exercise program will be led by a registered kinesiologist and will follow the Fundamental Lifestyle Medicine group class session. The program will be structured yet adaptable to accommodate each participant’s abilities and limitations. Before the exercise program, the kinesiologist will present the Education Sessions-Fitness Fundamentals, where they will explain why participants should exercise and how to be safe doing so. A booklet describing all activities will be shared with the participants that outline safe exercise guidelines, the rating of the Borg care of perceived exertion [44,55], when to stop exercising (symptoms and what to do), how to adapt the program, and exercises plan with picture of each exercise. The kinesiologist will then teach the entire exercise program in group sessions with the participants. Each session will last 1 hour and will follow this sequence warm-up, resistance training, cardio, flexibility, balance, core, and cool down. The exercise will use body weight resistance and a physiotherapy band appropriate for each person’s capacity.

#### Enrollment

Participants will be screened according to the inclusion and exclusion criteria by the physician during their initial medical consultation. Eligible participants will be informed about the study’s purpose, procedures, potential risks, and benefits during the informed consent process before enrolment. Participants will be made aware of their right to withdraw consent at any point during the study without any impact on their care.

#### Retention

The research staff and health care providers will strive to build strong rapport with participants encouraging them to attend the 22 weekly classes held in person at the local Parry Sound Bobby Orr Community Centre. If participants are unable to attend in person, a web-based platform option will be provided, along with access to recorded sessions. This approach ensured accessibility and adaptability to meet diverse patient needs. Attrition will be closely monitored, and for those who choose to leave the study, a structured exit interview with a research assistant will be conducted to gather feedback and identify potential areas for improving the program.

### Ethical Considerations

This study received ethics approval from the Laurentian University Research Ethics Board (6021397) on July 6, 2023, and adheres to the guidelines stated in the Declaration of Helsinki. This study was registered at ClinicalTrials.gov (NCT06192251) in November 2023. Trained research staff informed participants of their right to withdraw themselves and the information collected on them up until the time of withdrawal, and informed consent was obtained. Patient data is available only to program health care providers and is segregated from research data. Participants are assigned a research code following the informed consent process, which links their research data through the study to allow appropriate analyses. Participant information is deidentified, and results will be published in this manner as well to ensure confidentiality.

## Results

The first cohort of participants was enrolled in late 2023 and is still under evaluation. Data collection for the second cohort began in mid-2024 and is currently underway, with a projected end date in early 2025. A total of 16 participants have been recruited as of November 2024. Data analysis will be conducted in mid-2025, and we anticipate submitting the final manuscript by the end of 2025. A mixed method analysis [42,43] will be used to analyze the quantitative and qualitative data, collected individually at enrollment, program completion (22 weeks), and follow-up (6 months after program completion). Focus groups will be conducted after the 22-week intervention to assess the program’s effectiveness and implementation.

Initial findings indicate that participants have gained knowledge about lifestyle changes, particularly in stress management and health behavior choices, and positively impacted their friends, family, and community by sharing their experiences. As more data is analyzed, it is anticipated that participants who commit to making changes will show improvement in their physical and mental well-being with the knowledge and practices learned from the classes and interdisciplinary health team.

In addition, initial participants included members of the local First Nation communities who raised concerns about accessibility for other First Nations such as challenges with significant travel and limited access to web-based platforms. In response to these concerns, there is confirmed interest in hosting a CLIP-ON cohort within these communities to ensure equitable access for all interested members.

## Discussion

### Expected Outcomes

We anticipate that these findings will support the long-term goal of establishing a lifestyle medicine program for rural Ontario communities that combines education, digital platforms, and interactions with an interdisciplinary health team. Its holistic, patient-centered approach to medicine strives to promote lifestyle changes that can prevent and treat chronic diseases, transform patient care in a manner that has been demonstrated to be successful in large urban centers and encourage its adoption and adaptation by health care providers across Canada. Our long-term goal is to demonstrate that CLIP-ON positively impacts community health and decreases health care use by reducing the impact of chronic illness.

### Comparison to Previous Studies

CLIP-ON is the first lifestyle medicine program that will be conducted virtually and in-person in a rural Canadian community setting, while other Canadian lifestyle medicine interventions, such as Canadian Health Advanced Nutrition for Graded Exercise (CHANGE), have been implemented in large primary care settings in a physical format only [9,33]. This study will investigate the impact of incorporating all 6 pillars of lifestyle medicine as opposed to selected pillars [49-52,55-58]. It is also designed for patients with broad chronic diseases compared to other lifestyle medicine studies that focus on patients with specific chronic diseases [57,59-61]. Like the New York City lifestyle medicine program, CLIP-ON is built around all 6 pillars of health while providing individual support, goal setting, dietary recommendations, and monitoring support of an interdisciplinary health team [29,30,38]. However, Parry Sound’s catchment area spans 9222 km^2^ which is much larger than New York City’s 790-km^2^ urban setting [26,62]. The Parry Sound region includes 8 municipalities and townships and 4 First Nation communities housing over 42,000 residents who sometimes must travel long distances to access just primary care [26]. Therefore, understanding the unique challenges faced by rural Ontario communities will allow modifications to the program design that will be considerate of socioeconomic status, geographic and transportation barriers, preexisting patient-physician relationships, and cultural diversity.

The ability for participants to attend all programming virtually and in person was a core design to enhance program accessibility. This hybrid structure will also allow the onboarding of health care providers located throughout the province to engage in CLIP-ON more easily. Considering the vast catchment area of Parry Sound, limited transportation methods, and financial disparity within the region, it is understood that all participants may not have or have access to weekly transportation for classes. The research team will assist in identifying patient transport services to facilitate participant attendance at key program sessions wherever possible to enhance accessibility. A preexisting patient-physician relationship may result in discomfort for either participants or providers or introduce biased treatment towards some participants. Therefore, the research team will ensure that participants are matched with providers that they do not have an existing professional relationship with. Finally, special considerations will be incorporated for local Indigenous populations to be inclusive and respect their cultural requirements, including a land acknowledgment before every session and including providers with related experience and understanding of individual challenges and cultural differences.

These design elements distinctly position CLIP-ON as a whole health program designed to help all patients incorporate changes in various aspects of their lives in a setting where a lifestyle medicine program has yet to be introduced and piloted.

### Strengths

CLIP-ON is the first lifestyle medicine program designed for rural communities in Ontario. This included extensive engagement with local health care providers and community members to understand their unique challenges and preferences. It incorporates specific design elements such as virtual programming, which increases program accessibility for patients facing geographic barriers so that they can attend classes, exercise sessions, and meetings with health care providers remotely. The hybrid delivery model provides flexibility, allowing patients to choose in-person or virtual participation, which is crucial for geographically isolated individuals. In addition, it enables the recruitment of remote health care providers, increasing the feasibility of building an interdisciplinary health team for the program. This flexibility offers CLIP-ON to be a sustainable, impactful, and scalable model of preventative health care.

The involvement of an interdisciplinary team of health care professionals, including physicians, dietitians, health coaches, and kinesiologists, provides a comprehensive care approach that addresses various facets of participants’ health, enhancing the likelihood of sustainable health improvements. Furthermore, the program’s use of the RE-AIM framework ensures that implementation is evaluated through a robust and credible scientific approach, facilitating future scalability and applicability to other regions.

Initial observations have noted that the group structure and interactions during lifestyle medicine classes and exercise sessions facilitate social connectedness within the first cohort. The program’s focus on peer support and social interaction has led to increased participant accountability, as individuals share challenges and strategies within the group setting. Research shows that this peer support is often key to maintaining long-term behavior change [63]. Finally, the 2-cohort design of this study enables efficient incorporation of feedback from cohort one to enhance the program design for cohort two. The patient-centered approach, which integrates real-time feedback from participants and health care providers through focus groups and surveys, ensures continuous refinement of the program to meet participants’ needs and further enhance satisfaction and engagement.

### Limitations

This study has some limitations. Parry Sound’s small core town population of 6879 combined with the geographic barriers associated with its vast catchment area and lack of public transit limited the initial recruitment to only 8 participants in the first cohort, as opposed to the anticipated 10-12 [25]. This small sample size may limit the statistical power of the study, making it difficult to establish significant findings that are generalizable to other rural communities. To mitigate this, we are actively exploring strategies such as broader outreach to health care providers within the catchment area and forming partnerships with community organizations to raise awareness of the program.

Initial feedback also suggests that some participants found the program duration of 6 months to be too short for achieving and maintaining meaningful lifestyle changes. Extending the program duration could allow participants more time to solidify lifestyle adjustments. In addition, longer follow-ups will support the long-term impact of lifestyle medicine on chronic disease management. To facilitate this, additional follow-up sessions and group support beyond the 6-month mark are being established to reinforce lifestyle habits. This could offer a more gradual transition toward self-management for participants.

The web-based platform, while increasing accessibility for most, may present technological barriers for older participants or those unfamiliar with using web-based platforms. This could potentially reduce engagement for certain segments of the population, especially if support for technology use is not adequately provided. To alleviate this, we have introduced a brief training session for participants on how to use the virtual platform, and technical support is now available throughout the program.

### Future Directions and Dissemination Plan

We plan to conduct a follow-up assessment at 12 months and beyond to evaluate the sustainability of lifestyle changes and improvements in health outcomes among participants to provide valuable insights into the long-term impact of the CLIP-ON program. We will also explore the possibility of scaling the CLIP-ON program to other rural communities in Northern Ontario, considering adaptations based on the unique needs and cultural contexts of those populations. We will collaborate with local Indigenous communities to incorporate traditional health practices and teachings into the CLIP-ON program. This integration may enhance cultural relevance and improve health outcomes among Indigenous participants. We also plan to investigate the use of mobile health applications and web-based platforms to enhance participant engagement and accessibility such as tools for tracking progress, providing education resources, and facilitating communication with health care providers.

The results of this study will be shared locally through grand round presentations and with the hospital senior team and board members. There is a commitment from West Parry Sound Health Centre to support this study, and findings are regularly shared with senior leadership, the local education group executive, local primary care provider family health teams, and the Parry Sound Ontario Health Team. We will provide the results of the findings to each of these groups in an appropriate presentation at their request. In addition, we will share the results through publication and presentation within our Northern Ontario School of Medicine University and through presentation at the annual research conference.

### Conclusion

This protocol paper will provide valuable insights into the implementation of a lifestyle medicine program, which will be evaluated for its impact on participants’ health. The goal is to establish and disseminate an effective framework for secondary prevention, management, and in some cases reversal of common chronic diseases. By assessing the real-world implementation of this program, we aim to identify both successes and areas for improvement, ensuring the feasibility and sustainability of integrating lifestyle medicine into routine health care practices.

This comprehensive evaluation will not only guide future advancements in lifestyle medicine but also help establish a culturally inclusive and scalable model that can be adapted to benefit other communities, particularly those in resource-limited or rural settings.

## Appendix

Multimedia Appendix 1 CLIP-ON (Complete Lifestyle medicine Intervention Program–Ontario): Phase I Program Evaluation Plan (RE-AIM Framework).

## Abbreviations

CHANGE: Canadian Health Advanced Nutrition for Graded Exercise
CHIP: Complete Health Improvement Program
CLIP-ON: Complete Lifestyle medicine Intervention Program–Ontario
NCD: noncommunicable disease
P4: Preventive, Predictive, Personalized, and Participatory
PARmed-X: A Physical Activity Readiness Medical Examination
PAR-Q +: Physical Activity Readiness Questionnaire for Everyone
RE-AIM: Reach, Effectiveness, Adoption, Implementation, and Maintenance

## Data Availability

The datasets generated during the study are available from the corresponding author upon reasonable request.

## References

[1] Public Health Ontario, CCO (2019). The Burden of Chronic Diseases in Ontario.

[2] GBD 2019 DiseasesInjuries Collaborators (2020). Global burden of 369 diseases and injuries in 204 countries and territories, 1990-2019: a systematic analysis for the Global Burden of Disease Study 2019. Lancet.

[3] World Health Organization (WHO) Non communicable diseases.

[4] Sagner M, McNeil A, Puska P, Auffray C, Price ND, Hood L, Lavie CJ, Han Z, Chen Z, Brahmachari SK, McEwen BS, Soares MB, Balling R, Epel E, Arena R (2017). The P4 health spectrum - A predictive, preventive, personalized and participatory continuum for promoting healthspan. Prog Cardiovasc Dis.

[5] Alexander S, Ostfeld RJ, Allen K, Williams KA (2017). A plant-based diet and hypertension. J Geriatr Cardiol.

[6] Stauffer CM, McGlynn SM, Topor DR, Fiore L, Phillips EM (2022). Evaluation of a whole health-lifestyle medicine curriculum for physician assistant students: a mixed methods analysis. Med Sci Educ.

[7] Bodai BI, Nakata TE, Wong WT, Clark DR, Lawenda S, Tsou C, Liu R, Shiue L, Cooper N, Rehbein M, Ha BP, Mckeirnan A, Misquitta R, Vij P, Klonecke A, Mejia CS, Dionysian E, Hashmi S, Greger M, Stoll S, Campbell TM (2018). Lifestyle medicine: a brief review of its dramatic impact on health and survival. Perm J.

[8] Doughty KN, Del Pilar NX, Audette A, Katz DL (2017). Lifestyle medicine and the management of cardiovascular disease. Curr Cardiol Rep.

[9] Marin-Couture E, Filion MJ, Boukari R, Jeejeebhoy K, Dhaliwal R, Brauer P, Royall D, Mutch DM, Klein D, Tremblay A, Rhéaume C (2022). Relationship between cardiometabolic factors and the response of blood pressure to a one-year primary care lifestyle intervention in metabolic syndrome patients. Metabolites.

[10] Malecki HL, Gollie JM, Scholten J (2020). Physical activity, exercise, whole health, and integrative health coaching. Phys Med Rehabil Clin N Am.

[11] Government of Canada SC (2023). Diabetes among Canadian adults.

[12] van Ommen B, Wopereis S, van Empelen P, van Keulen HM, Otten W, Kasteleyn M, Molema JJW, de Hoogh IM, Chavannes NH, Numans ME, Evers AWM, Pijl H (2017). From diabetes care to diabetes cure-The integration of systems biology, eHealth, and behavioral change. Front Endocrinol (Lausanne).

[13] Khan MAB, Hashim MJ, King JK, Govender RD, Mustafa H, Al Kaabi J (2020). Epidemiology of type 2 diabetes - Global burden of disease and forecasted trends. J Epidemiol Glob Health.

[14] Espeland MA, Glick HA, Bertoni A, Brancati FL, Bray GA, Clark JM, Curtis JM, Egan C, Evans M, Foreyt JP, Ghazarian S, Gregg EW, Hazuda HP, Hill JO, Hire D, Horton ES, Hubbard VS, Jakicic JM, Jeffery RW, Johnson KC, Kahn SE, Killean T, Kitabchi AE, Knowler WC, Kriska A, Lewis CE, Miller M, Montez MG, Murillo A, Nathan DM, Nyenwe E, Patricio J, Peters AL, Pi-Sunyer X, Pownall H, Redmon JB, Rushing J, Ryan DH, Safford M, Tsai AG, Wadden TA, Wing RR, Yanovski SZ, Zhang P, Look AHEAD Research Group (2014). Impact of an intensive lifestyle intervention on use and cost of medical services among overweight and obese adults with type 2 diabetes: the action for health in diabetes. Diabetes Care.

[15] Sporinova B, Manns B, Tonelli M, Hemmelgarn B, MacMaster F, Mitchell N, Au F, Ma Z, Weaver R, Quinn A (2019). Association of mental health disorders with health care utilization and costs among adults with chronic disease. JAMA Netw Open.

[16] Mauriello L, Artz K (2023). Digital lifestyle medicine: designing, delivering, and scaling for impact. Am J Lifestyle Med.

[17] Phillips EM, Frates EP, Park DJ (2020). Lifestyle medicine. Phys Med Rehabil Clin N Am.

[18] American College of Lifestyle Medicine. Overview.

[19] CDC (2023). Health and Economic Costs of Chronic Diseases. CDC.

[20] McDonald A (2022). Incorporating lifestyle medicine into practice: a prescription for better health. Am Fam Physician.

[21] Klein D, Jeejeebhoy K, Tremblay A, Kallio M, Rheaume C, Humphries S, Royall D, Brauer P, Heyland D, Dhaliwal R, Mutch DM (2017). The CHANGE program: exercise intervention in primary care. Can Fam Physician.

[22] Kushner RF, Sorensen KW (2013). Lifestyle medicine: the future of chronic disease management. Curr Opin Endocrinol Diabetes Obes.

[23] Flores M, Glusman G, Brogaard K, Price ND, Hood L (2013). P4 medicine: how systems medicine will transform the healthcare sector and society. Per Med.

[24] Trapp CB, Barnard ND (2010). Usefulness of vegetarian and vegan diets for treating type 2 diabetes. Curr Diab Rep.

[25] Government of Canada SC (2022). Profile table, census profile, 2021 census of population. Parry Sound, Town (T) [Census subdivision].

[26] Government of Canada SC (2017). Census profile, 2016 census - parry sound, district [Census division]. Ontario and Ontario [Province].

[27] Chronic Diseases - North Bay Parry Sound District Health Unit.

[28] Redvers N, Blondin B (2020). Traditional indigenous medicine in North America: a scoping review. PLoS One.

[29] Albert SL, Massar RE, Kwok L, Correa L, Polito-Moller K, Joshi S, Shah S, McMacken M (2024). Pilot plant-based lifestyle medicine program in an Urban public healthcare system: evaluating demand and implementation. Am J Lifestyle Med.

[30] Babich JS, McMacken M, Correa L, Polito-Moller K, Chen K, Adams E, Morgenstern S, Katz M, Long TG, Joshi S, Wallach AB, Shah S, Boas R (2024). Advancing lifestyle medicine in New York City's public health care system. Mayo Clin Proc Innov Qual Outcomes.

[31] Curran GM, Bauer M, Mittman B, Pyne JM, Stetler C (2012). Effectiveness-implementation hybrid designs: combining elements of clinical effectiveness and implementation research to enhance public health impact. Med Care.

[32] Lifestyle Medicine Programs. NYC Health + Hospitals.

[33] Marin-Couture E, Moulin JA, Thibault AS, Poirier P, Després JP, Gallant A, Lamarre V, Alméras N, Lemieux I, Chabot C, Gallani M, Piché ME, Arsenault B, Tremblay A, Paquette JS, Rhéaume C (2024). Impact of lifestyle medicine interventions on the management of systemic hypertension in primary care: a Canadian randomized controlled trial. American Journal of Lifestyle Medicine.

[34] Lambrinou E, Hansen TB, Beulens JW (2019). Lifestyle factors, self-management and patient empowerment in diabetes care. Eur J Prev Cardiol.

[35] Wong VWH, Ho FYY, Shi NK, Sarris J, Ng CH, Tam OKY (2022). Lifestyle medicine for anxiety symptoms: a meta-analysis of randomized controlled trials. J Affect Disord.

[36] Buxton A (2022). NYC doctors to receive free plant-based nutrition training under $44M medicine program. Plant Based News.

[37] Parry sound, oN - demographics. Townfolio.

[38] (2024). Plant-Based Lifestyle Medicine Program. NYC Health + Hospitals.

[39] Dissemination Implementation.

[40] Glasgow RE, Harden SM, Gaglio B, Rabin B, Smith ML, Porter GC, Ory MG, Estabrooks PA (2019). RE-AIM planning and evaluation framework: adapting to new science and practice with a 20-year review. Front Public Health.

[41] Aim RE Home-reach effectiveness adoption implementation maintenance.

[42] Achieving integration in mixed methods designs-principles and practices - PubMed.

[43] Research Design.

[44] Foley M Canadian Society for Exercise Physiology (CSEP).

[45] Warburton DER, Jamnik VK, Bredin SSD, McKenzie DC, Stone J, Shephard RJ, Gledhill N (2011). Evidence-based risk assessment and recommendations for physical activity clearance: an introduction. Appl Physiol Nutr Metab.

[46] School of public health. Loma Linda University.

[47] 36-Item Short Form Survey (SF-36). RAND.

[48] The PHQ-9 - PMC.

[49] Shurney D, Hyde S, Hulsey K, Elam R, Cooper A, Groves J (2012). CHIP lifestyle program at Vanderbilt University demonstrates an early ROI for a diabetic cohort in a workplace setting: a case study. Journal of Managed Care Medicine.

[50] Shurney D, Hyde S, Hulsey K, Elam R, Cooper A, Groves J (2012). CHIP lifestyle program at vanderbilt university demonstrates an early ROI for a diabetic cohort in a workplace setting: a case study. Journal of Managed Care Medicine.

[51] Scottish Action for Mental Health Wellbeing Assessment. SAMH.

[52] Tennant R, Hiller L, Fishwick R, Platt S, Joseph S, Weich S, Parkinson J, Secker J, Stewart-Brown S (2007). The warwick-edinburgh mental well-being scale (WEMWBS): development and UK validation. Health Qual Life Outcomes.

[53] Keyes CLM (2002). The mental health continuum: from languishing to flourishing in life. J Health Soc Behav.

[54] Ainsworth F (2019). M. Seligman (2018). The hope circuit. A psychologist’s journey from helplessness to optimism. Sydney: Penguin Random. Children Australia.

[55] Borg Rating Of Perceived Exertion. Physiopedia.

[56] Jaqua E, Biddy E, Moore C, Browne G (2023). The impact of the six pillars of lifestyle medicine on brain health. Cureus.

[57] Walrabenstein W, Wagenaar CA, van der Leeden M, Turkstra F, Twisk JWR, Boers M, van Middendorp H, Weijs PJM, van Schaardenburg D (2023). A multidisciplinary lifestyle program for rheumatoid arthritis: the 'Plants for Joints' randomized controlled trial. Rheumatology (Oxford).

[58] Medawar E, Huhn S, Villringer A, Veronica Witte A (2019). The effects of plant-based diets on the body and the brain: a systematic review. Transl Psychiatry.

[59] Beetham KS, Krishnasamy R, Stanton T, Sacre JW, Douglas B, Isbel NM, Coombes JS, Howden EJ (2022). Effect of a 3-Year lifestyle intervention in patients with chronic kidney disease: a randomized clinical trial. J Am Soc Nephrol.

[60] Konieczna J, Ruiz-Canela M, Galmes-Panades AM, Abete I, Babio N, Fiol M, Martín-Sánchez V, Estruch R, Vidal J, Buil-Cosiales P, García-Gavilán JF, Moñino M, Marcos-Delgado A, Casas R, Olbeyra R, Fitó M, Hu FB, Martínez-Gonzalez MÁ, Martínez JA, Romaguera D, Salas-Salvadó J (2023). An energy-reduced mediterranean diet, physical activity, and body composition: an interim subgroup analysis of the PREDIMED-plus randomized clinical trial. JAMA Netw Open.

[61] Islam NS, Zanowiak JM, Wyatt LC, Chun K, Lee L, Kwon SC, Trinh-Shevrin C (2013). A randomized-controlled, pilot intervention on diabetes prevention and healthy lifestyles in the New York City Korean community. J Community Health.

[62] New York City (2024). Britannica.

[63] Latkin CA, Knowlton AR (2015). Social network assessments and interventions for health behavior change: a critical review. Behav Med.

