# Supplementary material for: Complete Lifestyle Medicine Intervention Program–Ontario: Implementation Protocol for a Rural Study
Source: JMIR Res Protoc. 2024 Dec 31;13:e59179. doi: 10.2196/59179 (PMC11733517; doi:10.2196/59179)
Supplement: Multimedia Appendix 1 [file resprot_v13i1e59179_app1.docx]

**CLIP-ON: Phase I Program Evaluation Plan (*RE-AIM Framework*)**

| **RE-AIM Component Definition** | **Quantitative & Qualitative Evaluation Components** |
| --- | --- |
| **Reach** |  |
| 1. Percentage of individuals who participate based on valid denominator (based on prevalence of chronic illness in rural population) | **Quantitative:**   - *Program-level*: Number of participants engaging in the CLIP-ON program relative to the total number of eligible participants living within the township (*denominator based population data*) - *Patient-level:* Number of physician-referred participants who engage in the CLIP-ON program relative to the total number of participants referred to the program (*denominator based on physician-tracked data*) - *Patient-level:* Number of self-referred participants who engage in the CLIP-ON program relative to the number of participants who inquire about the program   **Qualitative:**   - NA |
| 2. Characteristics of participants compared to nonparticipants or to target population | **Quantitative:**   - *Patient-level*: Medical, socio-demographic, behavioural characteristics assessed via program level survey and medical record extraction for consenting survey respondents   **Qualitative:**   - *Patient-level*: Individual preferences assessed via initial interview   - Lifestyle needs of respondents related to their current needs   - Reasons for decline of study participation in non-participants and reasons   for limited reach |

- Barriers and facilitators to study participation

| 3. Exclusion criteria | **Quantitative:**   - *Program-level*: Participants who do not meet inclusion criteria - *Patient-level*: Report numbers excluded by CLIP staff during baseline screening   **Qualitative:**   - NA |
| --- | --- |
| **Effectiveness** |  |
| 1. Measure of primary outcome: Feasibility (Loss to follow-up (<30%)) | **Quantitative:**   - *Patient-level*: Defined as completion rate of follow-up assessments   **Qualitative:**   - *Patient-level*: Reasons for loss to follow-up (using one-on-one participant interviews post-intervention) |
| 2. Measure of primary outcome: Intervention | **Quantitative:**   - *Patient-level*: Improved control over chronic illness (reduced reliance on medication, better patient reported quality of life)   **Qualitative:**   - NA |
| 3. Measure of broader outcomes: Feasibility (e.g., attendance, adherence) | **Quantitative:**   - *Program-level* (Program Capacity (≥80%)): Defined as the ratio of filled program spots to total program spots (i.e., total 20 program spots (2 cohorts sessions × 10 spots per session)) - *Patient-level* (Attendance ≥70%): Defined as ratio of total attended to total   planned sessions |

|  | - *Patient-level* (Intervention discontinuation): Defined as intervention drop-out prior to completion of all planned sessions - *Patient-level* (Intervention interruption): Defined as missing ≥3 consecutive   sessions   - *Patient-level* (Attendance and compliance with Nutritionist): Defined as positive changes made after regular attendance with individual appointments - *Patient-level* (Attendance and compliance with Health Coach): Defined as positive changes made after regular attendance with individual appointments - *Patient-level* (Adherence): Defined as compliance with the planned behavioral and physical recommendations prescribed by LM physician   **Qualitative:**   - Semi-structured one-on-one interviews will be conducted with participants post- intervention to determine:   - Reasons for feasibility outcomes   - Barriers and facilitators to program attendance and adherence   - Meaningfulness of efficacy outcomes   - Suggested improvements for future programming   - Further needs of participants related to exercise post cancer diagnosis |
| --- | --- |
| 4. Measure of broader outcomes: Intervention (e.g., improved physical abilities, quality of life) | **Quantitative:**   - *Patient-level*: Patient-reported Outcomes (PROs)   - *Patient-level*: Demographics (study-specific tool); Depression & Anxiety (RAND); Health-Related Quality of Life (HRQoL) (MOS-SF20); Lifestyle Medicine Survey   **Qualitative:**   - - Semi structured interview feedback |

| 5. Measure of broader outcomes: Safety (e.g., study-related adverse events) | **Quantitative:**   - *Patient-level*: Adverse Events   - *Intervention-Related:* Defined as the type, frequency, and severity of serious (e.g., important medical events) and nonserious adverse events (e.g., knee and back pain).   - *Non-Intervention-Related:* Defined as the type, frequency, and severity of serious (e.g., important medical events) and nonserious adverse events (e.g., knee and back pain).   **Qualitative:**   - NA |
| --- | --- |
| 6. Measure of robustness across subgroups | **Quantitative:**   - *Patient-level*: Comparison of all safety and efficacy outcomes between different chronic illness diagnosis groups   **Qualitative:**   - NA |
| 7. Measure of short-term attrition (%) and differential rates by patient characteristics or treatment condition | **Quantitative:**   - *Patient-level*: Comparison of all other intervention tolerability outcomes between different chronic illness diagnosis groups |

|  | **Qualitative:**   - *Patient-level:* Comparison of reasons for feasibility outcomes and barriers to program attendance and adherence between different chronic illness diagnosis groups (using one-on-one participant interviews post-intervention) |
| --- | --- |
| **Adoption** |  |
| 1. Percentage of settings approached that participated | **Quantitative:**   - *Setting-level*:   - Defined as the ratio of ‘providers who maintain collaboration/support’ to ‘providers’   *Staff-level*: Defined according to the quality and completeness of trial data collection and consistency of intervention delivery.  **Qualitative:**   - NA |
| 2. Characteristics of settings participating compared to either nonparticipants or some relevant resource data | **Quantitative:**   - *Setting-level*:   - Compare characteristics of ‘physicians/clinicians who maintain collaboration/ support’ to ‘total physicians/clinicians who offer collaboration/support’ - *Staff-level*: Not applicable at this phase   **Qualitative:**   - *Setting-level:* Reasons for continued collaboration of physicians/clinics who support the intervention (organizational survey) |

| 3. Setting exclusions (% or reasons) | **Quantitative:**   - *Setting-level*: Report the number and reasons for physical and clinic exclusions (*if applicable*)   **Qualitative:**   - *Setting-level*: Reasons for adoption or lack of adoption across targeted settings (organization survey) - *Staff-level:* Reasons for adoption or lack of adoption of data collection and intervention delivery protocol (staff focus group) |
| --- | --- |
| **Implementation** |  |
| 1. Percentage of perfect delivery or calls completed, and so on. (e.g., adherence or consistency) | **Quantitative:**   - *Program-level*: Defined as the ratio of ‘successful CLIP-ON programs delivered’ to ‘total number of CLIP-ON programs initiated’ - *Patient-level*: Patient adherence (*see Feasibility Outcomes*)   **Qualitative:**   - NA |
| 2. Adaptations made to intervention during study | **Quantitative:**   - *Patient-level*: Track and report modifications required for patients, and all other patient-level intervention changes (*see Feasibility Outcomes*)   **Qualitative:**   - *Program-level*: Track and report systemic intervention changes   - Reasons for adaptations/alterations made to intervention during the study considering staff/setting/time/subgroups/program delivery (staff focus group) |
| 3. Cost of intervention (time & money) | **Quantitative:** |

|  | - *Program-level*: Track and report costs associated with infrastructure development and maintenance - *Program-level*: Track and report costs associated with program delivery including materials, space, and human resources   **Qualitative:**   - NA |
| --- | --- |
| 4. Consistency of implementation across staff/time/ settings/subgroups (about process, not differential outcomes) | **Quantitative:**   - *Program-level*: Compare *Program-level* ‘Reach’ and ‘Adoption’ metrics between   cohorts   - *Patient*-level: Compare *Patient-level* ‘Reach,’ ‘Efficacy,’ and ‘Adoption’ metrics between (1) cohorts and (2) using *apriori*-defined patient characteristics   **Qualitative:**   - *Program-level*: Reasons for lack of consistency of implementation across staff/ time/settings/cohorts (staff focus group) |
| **Maintenance** (*Patient-level*) |  |
| 1. Measure of primary outcomes with ≥6-month follow-up after final program contact | **Quantitative:**   - 6-month follow-up survey and interview   **Qualitative:**   - Reasons for loss to follow-up (using one-on-one participant interviews at 6-month follow-up) |
| 2. Measure of broader outcomes or use of multiple criteria at follow-up | **Quantitative:**   - 6-month follow-up assessments of applicable fitness/functional/patient-reported outcomes   **Qualitative:** |

|  | - NA |
| --- | --- |
| 3. Measure of long-term robustness data | **Quantitative:**   - 6-month comparison of applicable Lifestyle Medicine patient-reported outcomes between different chronic disease conditions   **Qualitative:**   - NA |
| 4. Measure of long-term attrition (%) and differential rates by patient characteristics or treatment condition | **Quantitative:**   - 6-month comparison of feasibility outcomes between different patient populations   **Qualitative:**   - Semi-structured one-on-one interviews will be conducted with participants at 6- month follow-up to determine:   - Reasons for current level of commitment to Lifestyle Medicine and for all of the six pillars   - Current barriers and facilitators to participation   - Reasons why individual benefit continues or fades   - Meaningfulness of outcomes at 6-month follow-up |
| **Maintenance** (*Program-level*) |  |
| 1. Potential for program uptake | **Quantitative:**   - NA   **Qualitative:**   - Discussion and evaluation of alignment between CLIP and WPSHC missions and appraisal of potential ongoing institutional support for the CLIP program |

- Reasons why the organization delivering the intervention decides to continue or discontinue the intervention (to determine sustainability) (organization focus group at end of study period)
